# Evaluation of Flexible Force Sensors for Pressure Monitoring in Treatment of Chronic Venous Disorders

**DOI:** 10.3390/s17081923

**Published:** 2017-08-21

**Authors:** Suresh Parmar, Iryna Khodasevych, Olga Troynikov

**Affiliations:** School of Fashion and Textiles, Royal Melbourne Institute of Technology, Melbourne 3056, Australia; s3524484@student.rmit.edu.au (S.P.); iryna.khodasevych@rmit.edu.au (I.K.)

**Keywords:** pressure sensor, sensor evaluation, calibration, force-sensing, low interface pressure, compression therapy, piezoresistive sensors

## Abstract

The recent use of graduated compression therapy for treatment of chronic venous disorders such as leg ulcers and oedema has led to considerable research interest in flexible and low-cost force sensors. Properly applied low pressure during compression therapy can substantially improve the treatment of chronic venous disorders. However, achievement of the recommended low pressure levels and its accurate determination in real-life conditions is still a challenge. Several thin and flexible force sensors, which can also function as pressure sensors, are commercially available, but their real-life sensing performance has not been evaluated. Moreover, no researchers have reported information on sensor performance during static and dynamic loading within the realistic test conditions required for compression therapy. This research investigated the sensing performance of five low-cost commercial pressure sensors on a human-leg-like test apparatus and presents quantitative results on the accuracy and drift behaviour of these sensors in both static and dynamic conditions required for compression therapy. Extensive experimental work on this new human-leg-like test setup demonstrated its utility for evaluating the sensors. Results showed variation in static and dynamic sensing performance, including accuracy and drift characteristics. Only one commercially available pressure sensor was found to reliably deliver accuracy of 95% and above for all three test pressure points of 30, 50 and 70 mmHg.

## 1. Introduction

Chronic venous disorders such as leg ulcers, oedema and venous stasis reduce the quality of life of patients worldwide by impacting their physical, emotional and financial well-being [1,2,3]. Compression therapy—the application of persistent pressure on the surface of a limb, most often a leg—is a widely accepted clinical treatment for chronic venous disorders [4]. However, according to World Union of Wound Healing Societies’ consensus document [5] and the RAL-GZ 387-1 compression hosiery standard [6], compression bandages generally apply pressure below 70 mmHg for treatment of chronic venous leg ulcers. The recommended pressure can be further categorised, according to the pressure produced at ankle height, as mild (<20 mmHg), moderate (≥20–40 mmHg), strong (≥40–60 mmHg) or very strong (≥60 mmHg) [7]. Moreover, for a more pronounced effect, graduated compression therapy [8,9] that moderately reduces the allowed compression pressure range from ankle to calf is practiced. For instance, pressure of 20 mmHg (100%) at the ankle is reduced to 14–20 mmHg (70–100%) at position B1 (the transition point on the leg where the medial gastrocnemius muscle turns into its tendinous part) and to 10 mmHg (50%) at the calf. Researchers have measured the pressure applied to the leg using medical pressure measurement devices such as the Kikuhime^®^ [10,11,12,13], SIGaT-Tester^®^ [14], Picopress^®^ [14,15] and Pliance X^®^ [16] apparatus in clinical settings. However, being specialised medical devices, these instruments are very expensive and can only be used in a medical setting under the guidance of a trained medical attendant [17]. As a result, initiatives to develop a low-cost and reliable pressure monitoring device for medical applications are in progress [18,19,20,21,22,23]. In addition, research is required to simplify this pressure measurement process in compression therapy and provide flexibility to users/patients so they can self-administer bandages/hosiery. The development of devices for measuring the interface pressure generated by bandages and pressure garments usually follows the recommendations set out in the International Compression Club consensus paper [13,24,25]. According to these recommendations, an ideal interface pressure measurement device for medical application is small, thin, flexible, durable and reliable in an operating range consistent with biological parameters. Most commercially available pressure and force sensors were deemed unsuitable [26] due to inability to measure very low pressure levels, excessive thickness and being enclosed in a rigid metallic casing.

In recent years, thin, flexible force sensors such as the Peratech QTC™ [27], Interlink FSR^®^ [28], Sensitronics^®^ [29], Tactilus^®^ [30] and Tekscan Flexiforce^®^ [31] have appeared and been used as pressure sensors when the force is applied on their known surface area. This has found applications in fields such as robotics [32,33,34], sport [26,35,36], bioengineering [37,38] and medicine [39,40,41,42,43]. Force sensing resistors (FSRs) are suitable for medical applications due to their thin and flexible construction and ability to operate at low pressures. In addition, they are suitable for integration into clothing as well as for future wireless applications. They have lower accuracy than other types of force sensors, but seem more suitable for use in compression therapy because of their small size and thickness, low cost, easy customisation, and integration ability in textiles structures such as bandages and hosiery. While there are other pressure sensing technologies, not based on resistance change measurement, many of them are not in sufficient maturity state for wide practical application beyond laboratory. Although some studies [13,44,45] have determined the characteristics of FSRs for the aforesaid applications, no single study has provided a comprehensive comparative evaluation of all available sensors under the same test conditions. Further, it is standard practice to conduct measurements on sensors placed on a flat rigid surface, which is very different from placement on a human leg. No previous studies have reported information on sensor performance during static and dynamic loading within the realistic leg-like test conditions required for compression therapy. In addition, although it is widely believed that the stiffness and curvature of the human leg influences sensor properties and the interface pressure [4], no experiments on a soft surface that mimics human leg for compression therapy application have been published.

This paper presents a comparative performance evaluation of five commercially available low-cost piezoresistive sensors with potential application in compression therapy. The pressure measurements were conducted in a low pressure range of 20–70 mmHg appropriate for the interface pressure between a user’s limb and a pressure-inducing garment/bandage. An experimental pressure measurement system, its devices and circuitry, as well as the performance parameters required for effective validation of the interface device, were developed in this study. Parameters such as accuracy, static drift, dynamic drift and repeatability were defined for common evaluation of sensors. The sensor output was also compared with a weight-based ‘gold standard’ to determine the accuracy of these sensors.

## 2. Materials and Methods

### 2.1. Sensors

**Figure 1:**
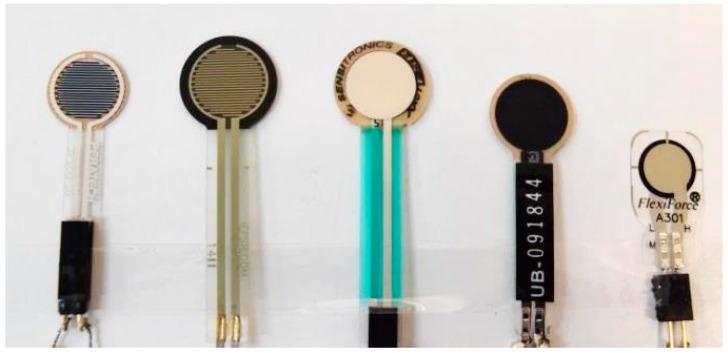
Evaluated force sensing resistors (left to right): Peratech QTC™, Interlink FSR^®^, Sensitronics^®^ FSR, Tactilus^®^ Sensor and Tekscan Flexiforce^®^ A301.

Commercially available thin and flexible piezoresistive force sensors were sourced for this study. These sensors were single-contact point force sensors, and so were able to provide a single sensing pressure output based on the area of applied load. The conductivity of these piezoresistive sensors rises with applied pressure due to increased contact between conductive particles in the polymer matrix of the sensor. The five sensors evaluated were the Peratech QTC™, FSR^®^, Sensitronics^®^, Tactilus^®^ and Flexiforce^®^ (Figure 1).

The Peratech QTC™ sensor is manufactured by Peratech Holdco Ltd. (Richmond, UK) from a pressure-sensitive quantum tunnelling composite (QTC™). The Peratech SP200 series utilized in this study is comprised of silver ink-based electrical connections that extended into carbon ink-based finger electrodes. A QTC patch is assembled over the finger electrodes with a 50 μm adhesive layer surrounding the active area. This creates a gap between the top electrode and the QTC layer [27]. The QTC composite comprising nickel powder filler in an elastomer matrix is prepared by a patented process [46] that involves the careful mixing of fillers and liquid monomers. The fillers possess sharp surface protrusions so that the electric field strength at their tips is very large and results in field assisted (Fowler–Nordheim) tunneling [47]. In addition, the filler particles are intimately coated by the polymer matrix leading to the particles not coming into direct physical contact with each other. The resultant composite enables a significant piezoresistive effect, due to the resistance of the composite being extremely sensitive to deformation. When compressed into the low-resistance state, the composite can carry large currents without observable damage. The Interlink FSR^®^ (Interlink Electronics Inc., Camarillo, CA, USA) is a thin-film device consisting of two conducting inter-digitated patterns printed on a polyester (Mylar) substrate that faces another substrate with a printed piezoresistive ink. These substrates are separated by a spacer that creates a gap when in an unloaded state and an active conduction path in a loaded state; they respond to a change in force with an approximately linear change in resistance. The Sensitronics^®^ FSR sensor (Sensitronics LLC, Bow, WA, USA) has similar construction, with two conductive silver paths separated by a spacer and pressure-sensitive film. The Tekscan^®^ Flexiforce A301 sensor (Tekscan Inc., South Boston, MA, USA) is constructed of two layers of polyester substrate; conductive silver is applied on each layer, followed by a layer of pressure-sensitive ink. Finally, the Tactilus^®^ sensor (Sensor Products Inc., Madison, NJ, USA) incorporates screen-printed resistive ink to separate the conductive paths. The geometry and the sensing properties of the sensors evaluated in this study are presented in Table 1.

### 2.2. Experimental Setup

**Figure 2:**
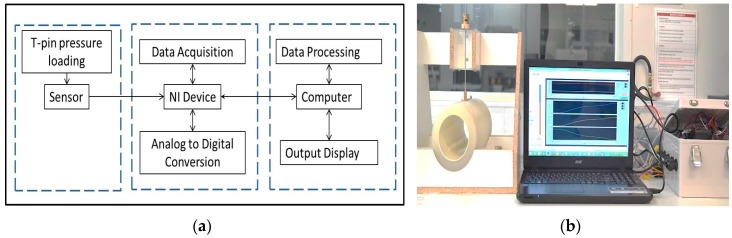
Experimental setup: (**a**) Block diagram; (**b**) Image of test setup.

**Figure 3:**
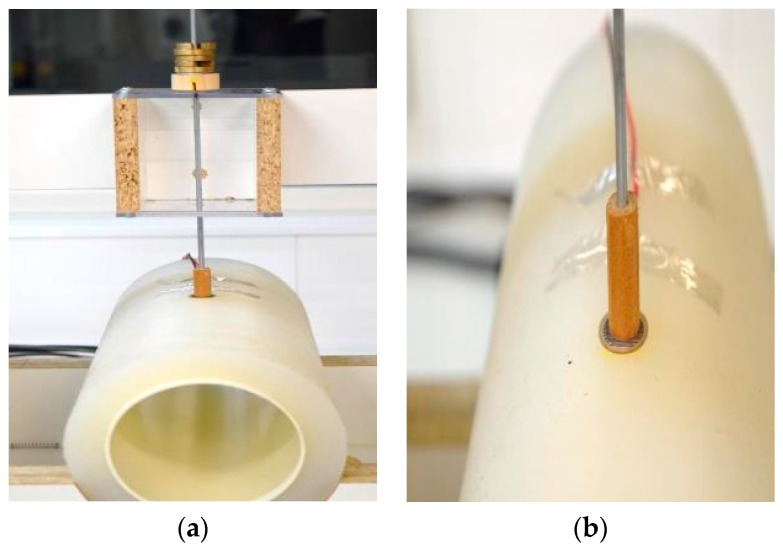
(**a**) Test setup with soft leg like silicone structure; (**b**) Placement of the sensor and t-pin.

In this study, the evaluation and determination of sensor properties was performed in a manner representative of the actual pressure measurement conditions on a human leg in compression therapy, using a custom-designed instrumental setup (Figure 2a,b). To mimic the flesh of a human leg, a soft and flexible core-sheath structure was developed as the surface of the compression testing apparatus (Figure 3a).

To mimic the curvature of the human leg, the testing apparatus in this protocol consisted of a soft cylinder with the same circumference as a human calf [48]. The cylinder used in this protocol had a total diameter of 13 cm, consisting of a 2 cm thick sheath made from soft translucent silicone on a PVC cylinder of 9 cm diameter. Animatronic-grade silicone was sourced from Dalchem Chemicals (Cheltenham, VIC, Australia). The choice of the silicone, its stiffness and its thickness was based on earlier research [49], in which it was demonstrated that silicone with thickness of 2 cm has stiffness similar to that of human soft tissue. Furthermore, surface roughness of the silicone coated test cylinder was determined using Kawabata surface tester as described in [50,51] and was 0.1 μm with standard mean deviation being 0.008 μm. Therefore, the roughness of the cylinder’s surface for the purpose of the present research is considered to be negligible and not to be affecting the sensor performance. The cylinder was then fixed in a horizontal position on a wooden frame as shown in Figure 3a. A 1 cm × 1 cm test bed spot was marked on the top surface of the cylinder, and the sensors affixed to it using transparent adhesive tape. The adhesive tape was attached only to the electrodes and did not cover the sensing area.

In this custom-designed setup, pressure was applied to the sensor via a t-pin that had a platform to hold standard commercial deadweights on its top. The wooden t-pin had a flat bottom with a diameter of 9.4 mm so that all the sensors could be tested with the same t-pin (Figure 3b). A uniform pressure distribution on the sensor surface was maintained throughout the test by using a flat bottom of the t-pin and by restricting the motion of t-pin in a vertical direction using suitably fit grooves in the holder of the t-pin. In addition, the placement of the sensor and the experimental equipment was levelled using a tri-axial bubble spirit level so that a uniform pressure is applied on the sensor. Dead weights were slotted disks weighing 10 g each. Application of deadweights is one of the numerous methods of pressure application. It provides the benefit of consistent and repeatable pressure due to standardised weight. The application of pressure is nearly immediate once the weight is placed on the sensor, which is beneficial under dynamic testing conditions. It is also consistent with recommendation by the sensor manufacturers that the area over which pressure is applied is slightly less than the sensor’s physical dimension. The pressure exerted by weights on the sensor was calculated beforehand and was increased in a systematic manner as shown in Table 2. The pressure from the weight was calculated as:

(1) $$\mathrm{Applied}\;\mathrm{pressure}\;(\mathrm{mmHg})\;=\;\frac{\mathrm{force}}{\mathrm{area}}=\;\frac{\mathrm{weight}\;(g)\;\times 735.5591}{{\mathrm{contact}\;\mathrm{surface}\;\mathrm{area}\;(\mathrm{cm}}^{2})\times 1000}$$

This novel t-pin setup with dead-weights acted as the gold standard for comparison of the outputs of the sensors. The sensors were connected to RMIT University’s proprietary National Instrument-based electronic setup, run via LabView and incorporating automated data logging and pressure measurement software. All sensors were connected using 10 kΩ resistors in order to create a voltage divider and reduce supplied voltage to an acceptable value. Although the resistor with same resistance value was used for all sensors in order to maintain consistency in the experimental work, this also resulted in different output voltages for different sensors. However, output voltage is only linked to the resolution of the sensor and so can be adjusted by changing the value of the resistor connected to the sensor. The larger the output voltage, the smaller the increments of applied pressure the sensor can resolve. The acquired sensor output voltage signal was multiplexed, amplified, and passed through an analogue-to-digital converter to the computer. RMIT’s custom-designed software interpreted this raw data in real time and stored the raw data file to the computer. The raw voltage data was then pre-processed to determine corresponding pressure values based on calibration before analyzing for the various sensing parameters. All the sensors were subjected to the same series of static and dynamic tests and the corresponding pressure sensing parameters were derived and evaluated. In this study, a sensor was tested three times for dynamic studies to accommodate intra-sensor repeatability evaluation. Standard room temperature of 20 °C and relative humidity of 65% RH were maintained throughout the tests.

### 2.3. Method

Before testing, each sensor was conditioned five times dynamically for about 3 s by compressing to a pressure of approximately 80 mmHg through the t-pin so as to bring the sensor from a relaxed state to an active state. Deadweights were added to the t-pin platform to perform both calibration and loading tests. Further, to reduce variation due to manual testing, the t-pin was dropped from a height of 2 mm using a lever and was not allowed to rotate during the testing process. At first, each sensor was custom calibrated to allow analysis of the accuracy and repeatability of measurements. Subsequently, static and dynamic loading tests were performed to derive pressure sensing parameters. The process of calibration and loading tests is illustrated below.

#### 2.3.1. Sensor Calibration

**Figure 4:**
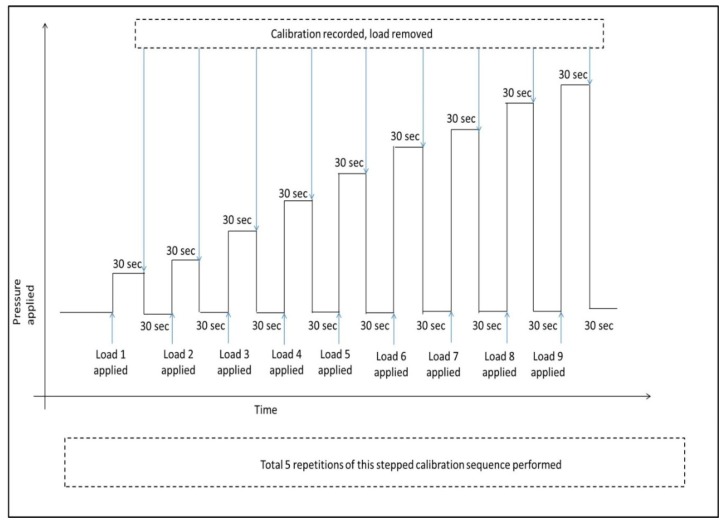
Loading sequence: Calibration cycle.

Prior to performing the loading tests, the sensor output voltage was referenced to known values of pressure applied to the sensor. Every sensor was calibrated individually by applying the weights presented in Table 2 up to 94.03 mmHg, in the loading sequence shown in Figure 4.

The stepped calibration procedure for raw data collection was as follows: The sensor output recording was started and data was acquired for 100 s without the load; the weight was applied on the sensor and the reference sensor voltage for the particular pressure recorded after 30 s. Then, the weight was removed for 30 s and the next sequentially stepped weight was applied for 30 s. In a real-life compression therapy scenario, a medical attendant applies the compression bandage and waits for a few seconds before noting the pressure readings, so that any variation in pressure due to changes in bandage application can be accommodated. Therefore, in this study the calibrations were recorded after 30 s. In addition, these intervals provide an important time gap for the sensors to reach a more stable condition, such that any change after this time could be attributed to drift. The whole calibration procedure for an individual sensor was repeated five times, and the software then calculated an average of these five calibration curves to give a single curve. RMIT’s customised software was programmed to automatically process this calibration data and remove outlier readings in each cycle of raw calibration file while creating the averaged calibration file for use in the pressure loading stage.

#### 2.3.2. Loading Tests

In this stage, the referenced values stored for a particular sensor in the processed average calibration data file were called in, calculated and displayed by the software in response to an applied pressure. The known applied pressure forms a base to determine the accuracy of the sensor’s output. In contrast, when an unknown pressure is applied on the sensor, this method enables calculation of pressure values. For this study, three pressures in the 20–70 mmHg range of compression therapy—30 mmHg, 50 mmHg and 70 mmHg—were selected, and the nearest pressure points in our dataset—30.5 mmHg, 51.4 mmHg and 72.7 mmHg—used for loading tests and evaluation of the five sensors. The loading sequence for static and dynamic tests was then performed to determine sensing parameters in both static and dynamic conditions. The parameters and the specific process to derive them are detailed below.

• Static Evaluation

Static measurements mimic prolonged wear of a compression garment under a constant pressure and were performed via separate applications of 30.5, 51.4 and 72.7 mmHg, and recording the pressure readings in the software. The pressure on the sensor was applied using the weights for 8 h, and the pressure measurements were simultaneously recorded by the RMIT software. The weight was then removed and the sensor allowed to relax for at least 2 h before commencing the next test with a new weight, corresponding to the next pressure level. This procedure was repeated until all the desired pressure levels were measured for every sensor.

• Dynamic Evaluation

**Figure 5:**
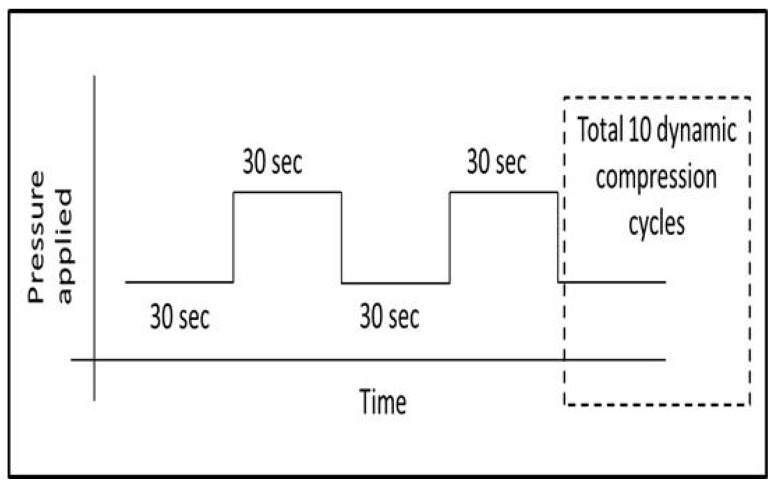
Loading sequence: Dynamic loading cycle.

Dynamic measurements mimic short-term changes in pressure during bandage application as well as during human physical activity. The three selected pressures (30.5, 51.4 and 72.7 mmHg) were periodically applied to the sensor placed on the soft cylinder surface. The dynamic loading cycle was initiated after collecting data for noise analysis by recording the sensor output without applying any load for 100 s. This relaxed recording stage was followed by dynamic loading, involving applying a cyclic load on the sensor with a holding time at each step of 30 s and a duty cycle of 50% (period = 60 s). This cycle was repeated 10 times (Figure 5). Dynamic cyclic testing (10 cycles) was repeated three times, after full relaxation of the sample for 30 min, to determine any repeatability issues due to relaxation in the sensor.

Subsequently, sensing parameters such as accuracy, voltage, pressure and voltage and pressuredrift, average pressure reading accuracy for static measurements and accuracy, voltage, pressure, average pressure readings accuracy and repeatability for dynamic measurements, of the sensor were calculated and graphically presented and analysed. The guidelines for parameter calculations are presented below.

### 2.4. Sensing Parameters and Data Analysis

The pressure sensing parameters and the methods adopted to derive those parameters for an effective comparison of sensors were as follows:

• Accuracy

This parameter demonstrates the closeness of the pressure measurements from a sensor to the actual applied pressure. Accuracy error (*AE*) in pressure measurement was calculated as a percentage based on the equation:

(2) $$AE=100\;(P_{l}-P_{s})/P_{l}$$

where *P_l_* is the pressure applied by the t-pin platform with the weights and *P_s_* is the corresponding referenced pressure for the sensor recorded by the software. *AE* is positive if the sensor reading is lower than the applied pressure and negative if the sensor reading is higher than the applied pressure. Then, the accuracy of the sensor at any moment in time was calculated as:

(3) A(t)=100−|AE(t)|

where absolute value of accuracy error *AE* is deducted from 100%. Accuracy of a sensor was determined for both static and dynamic pressure application profiles.

• Drift

Drift in a pressure sensor is defined as the change in output signal over a period of time independent of the applied pressure. Drift can be estimated for voltage and pressure parameters by comparing the final parameter value with the initial parameter value shortly after pressure application, but allowing sufficient time for rate of change of output parameter to reduce enough to be considered steady state (taken at 26 s after pressure application in this study). Drift, as a percentage of initial value, is determined as follows:

(4) $$D_{V}=100(Vf-Vi)/Vi$$

(5) Dp=100(Pf−Pi)/Pi

where *V_f_* and P_f_ are voltage and pressure at the final moment over which the pressure is applied, and *V_i_* and *P_i_* are voltage and pressure at the initial moment in time when rate of change of the output has reduced sufficiently to be considered steady state. Twenty-six seconds initial time was selected, as all sensors reach a steady state by this time. Drift was calculated for static measurements at 1 min, 5 min, 10 min, 1 h, 4 h and 8 h from the time of pressure application.

• Repeatability

The repeatability of the output of the sensor was determined by comparing the average pressure accuracy values and absolute deviations of three sets of dynamic tests.

## 3. Results

### 3.1. Static Evaluation

**Figure 6:**
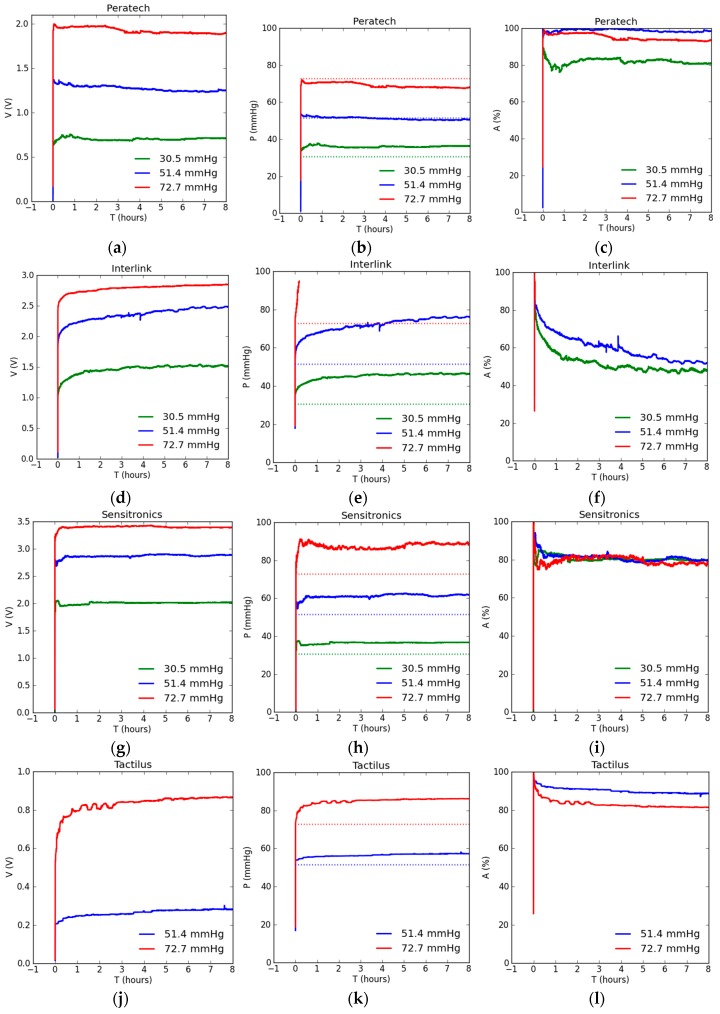

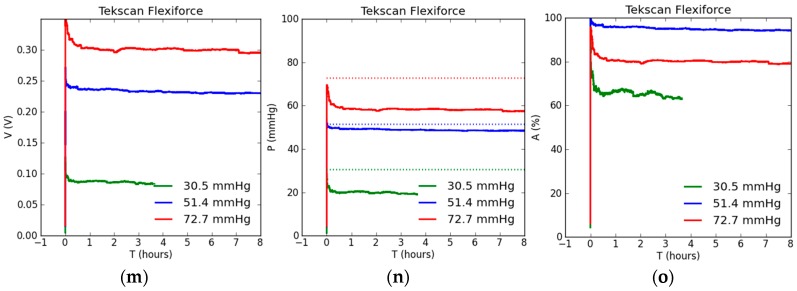
Static testing results for sensors: (**a**,**d**,**g**,**j**,**m**) Sensor output voltage reading; (**b**,**e**,**h**,**k**,**n**) Sensor pressure readings. Dotted lines show corresponding applied gold standard pressures of 30.5, 51.4 and 72.7 mmHg; (**c**,**f**,**i**,**l**,**o**) Accuracy of the sensor pressure readings.

Figure 6 shows experimental results for static testing of 8 h on all the sensors. Figure 6a,d,g,j,m shows output voltage results for the corresponding weights applied to the sensors.

The Sensitronics and Interlink sensors show the highest output voltages up to 3.5 V, whereas the Flexiforce and Tactilus ones show the lowest output voltages below 1 V. Output voltage represents the inherent sensor response unaffected by the choice of calibration method, and can be viewed as a baseline for the best achievable sensor performance. It can be seen from Figure 6 that all sensors exhibit drift in output voltage with time due to readjustment of the particles within the piezoresistive polymer as well as the viscoelastic nature of the polymer substrate. Most sensors show upward drift in voltage. Although the changes in pressure readings were observed for the whole period of pressure application, the changes due to drift seem to reduce significantly after the sensors attain a steady state. The sensors required different times to reach this steady state: the Peratech, Sensitronics and Flexiforce units achieved steady state the fastest, generally within a few minutes, while the Tactilus and Interlink devices required nearly an hour to reach the steady state.

Figure 6b,e,h,k,n shows pressure results obtained with reference to the calibration file, and demonstrates that they are affected by the calibration method and its accuracy. Pressure readings follow the same drift patterns displayed by output voltage readings. The larger the number of calibration points and the number of calibration readings at each pressure point, the greater the similarity between the pressure and voltage outputs of the sensor. However, there can still be differences caused due to inherent pressure vs voltage non-linearity in sensor output, noise in the measurement system and repeatability of the sensor response between consecutive measurements. Comparing pressure readings to actually applied gold standard pressures of 30.5, 51.4 and 72.7 mmHg, shown by the dotted lines, reveals that the most accurate pressure readings were achieved for all sensors, except Interlink, at an intermediate value of 51.4 mmHg. The Tactilus sensor exhibited no response at 30.5 mmHg of applied pressure, suggesting it has a threshold pressure below which it does not operate. Tekscan Flexiforce sensor readings were less than applied values for all pressures, Tactilus, Interlink and Sensitronics measured values were larger than applied for all pressures, and Peratech exhibited higher measured values for lower pressures, lower measured values for high pressures and very accurate values for medium pressure. It was also observed that at the loading test point of 72.7 mmHg, the voltage readings for Interlink sensor had drifted beyond the maximum calibrated pressure values within 20 min of applied load. It subsequently displayed only a straight line representing the maximum calibrated pressure reading of 94.03 mmHg and hence in Figure 6e,f the curve is presented only till the point where the curve showed calibrated pressure measurements. In contrast, pressure readings at 30.5 mmHg for Tekscan Flexiforce sensor drifted below the minimum calibrated pressure readings of 17.63 mmHg after about 3.5 h and so in Figure 6m,n,o only the reading till the point where the curve showed calibrated pressure measurements is shown.

Figure 6c,f,i,l,o shows the accuracy of the sensor pressure readings as a percentage of the gold standard represented by the applied weight. Accuracy is usually the practical parameter that determines the utility of a sensor in any application. For all tested sensors, except Sensitronics, accuracy of pressure readings depended on the amount of the applied pressure and reduced with time, eventually reaching steady state. Sensitronics was the only sensor to show a consistent level of accuracy at 80% for all tested pressure values. Accuracy levels were consistently highest at 51.4 mmHg of pressure. Peratech and Tekscan Flexiforce achieved over 95% accuracy at this level, with other sensors reaching 80–90% and Interlink as low as 60%. The highest pressure level of 72.7 mmHg was associated with an intermediate level of accuracy, predominantly around 80%, with Peratech at 95%. Measurements at the lowest pressure level of 30.5 mmHg were the least accurate, ranging between 55% and 80%. The results are summarized in Table 3. The most accurate readings were recorded shortly after application of pressure; this can be attributed to the calibration method. During calibration, pressure was applied for only 30 s before the reading was taken. Although this time was sufficient to achieve short-term steady state, it cannot compensate for drift in the readings over hours. For most sensors, accuracy of measurements approached a more stable state after about an hour after the pressure was first applied. Tekscan Flexiforce achieved a steady accuracy level within half an hour, and Interlink (at 72.7 mmHg) and Peratech (at 51.4 and 72.7 mmHg) achieved it even within a few minutes. However, Interlink displayed significant drift in accuracy for hours at 30.5 and 51.4 mmHg. As all the sensors achieve steady state, accuracy can be significantly improved by selection of calibration time close to the desired usage time of the sensor, and is not an inherent issue of the sensors.

**Figure 7:**
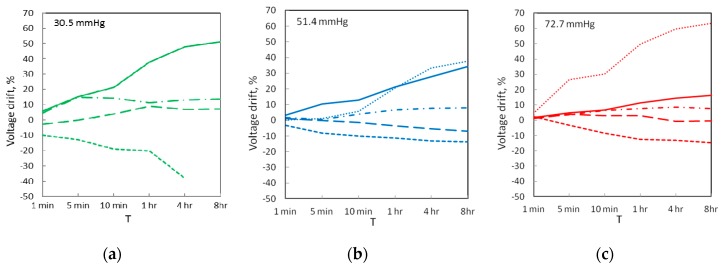

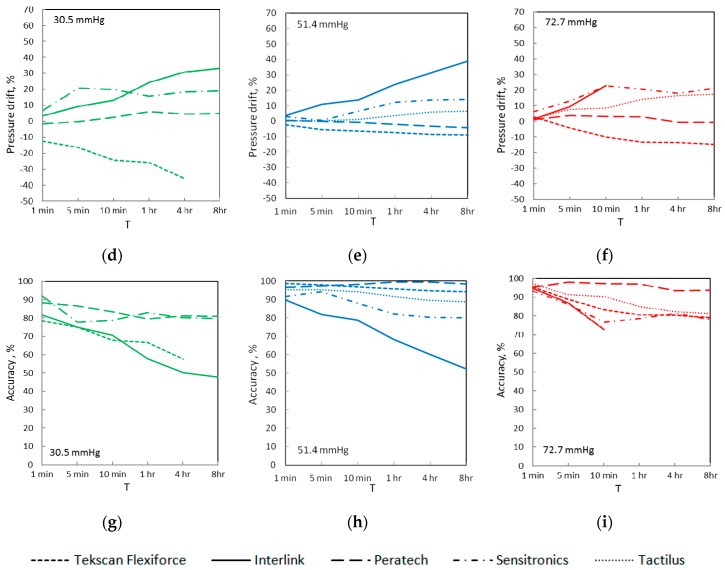
Static drift results for sensors at the three applied pressures: (**a**–**c**) Output voltage drift; (**d**–**f**) Pressure reading drift; (**g**–**i**) Accuracy of the sensor pressure readings.

Figure 7a,b,c shows output voltage drift for all sensors over a period of 8 h, as a percentage of initial value measured 26 s after the application of pressure. At 8 h the Sensitronics and Peratech sensors drifted the least, with drift values under 10% at all pressures. The Interlink and Tactilus sensors drifted the most, up to 50%. Larger drift values are a result of slower response, since initial voltage values are significantly lower than corresponding values for faster-responding sensors. All sensors display saturation on the curves due to reduced rate of change and steady-state operation. Drift in output pressure readings, shown in Figure 6d,e,f, generally follows voltage drift and is 10–20% for fast-responding sensors and up to 40% for slower-responding sensors.

Accuracy comparisons for all the sensors are shown in Figure 7g,h,i and Table 3. The best results were achieved at 51.2 mmHg for Peratech, followed by Tekscan Flexiforce and Tactilus. At 30.5 mmHg, Peratech and Sensitronics achieved better accuracy than other sensors, which was, however, lower than at 51.2 mmHg. Accuracy at 72.7 mmHg was intermediate between medium and lower pressure levels. Noise in the measurement system was also estimated from the results. In the absence of any applied pressure, noise level in the system was on the order of 10^−5^ V; with the pressure applied to the sensors, noise levels increased to the order of 10^−3^ V, which would be the resolution limit for the system.

### 3.2. Dynamic Evaluation

**Figure 8:**
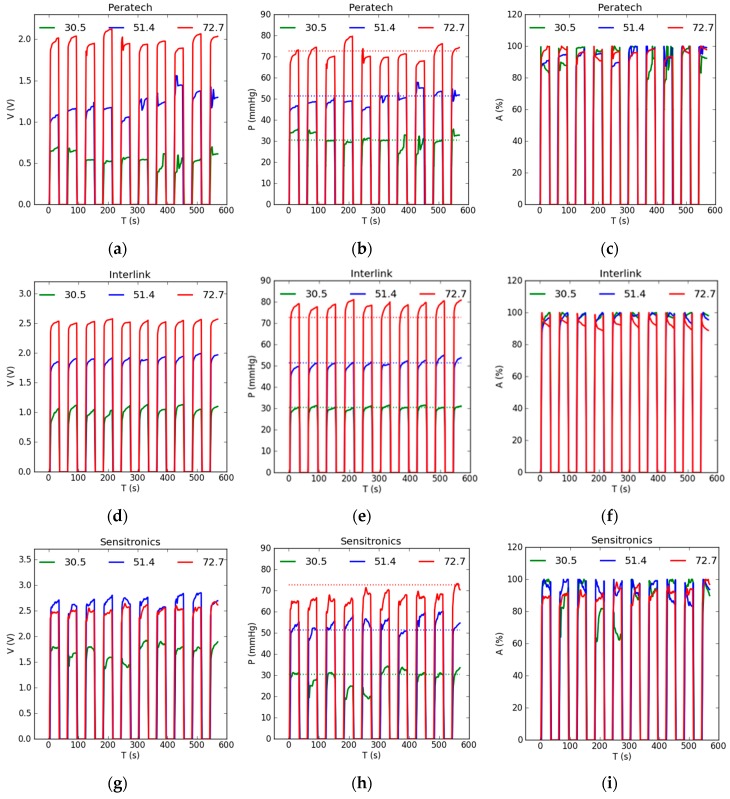

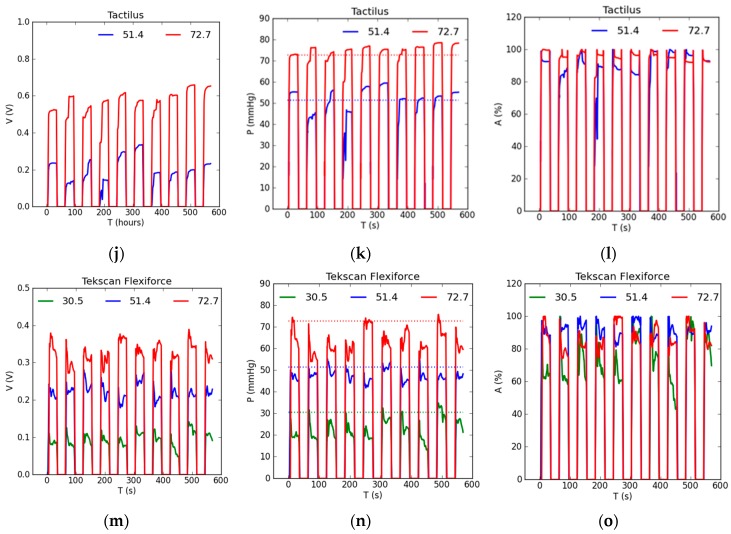
Dynamic testing results for sensors: (**a**,**d**,**g**,**j**,**m**) Sensor output voltage readings; (**b**,**e**,**h**,**k**,**n**) Sensor pressure readings. Dotted lines show corresponding applied gold standard pressures of 30.5, 51.4 and 72.7 mmHg; (**c**,**f**,**i**,**l**,**o**) Accuracy of sensor pressure readings.

Figure 8 shows experimental results for dynamic testing of the sensors. Figure 8a,d,g,j,m shows output voltage results. Two characteristics can be distinguished from the plots: the drift in output readings while the pressure is applied, and the repeatability of output readings between cycles. The Tekscan Flexiforce showed erratic behaviour within the cycle, with predominant downward drift consistent with the static case, and poor repeatability, especially for lower pressure of 30.5 mmHg. Other sensors showed predominantly upward drift within the cycle with occasional erratic behaviour; drift amounts relative to output values were similar for most sensors and pressure levels. The Peratech again was the best-performing sensor for dynamic operation, followed by the Sensitronics. The Interlink sensor displayed exceptional consistency in sensor behaviour during pressure application, as well as high repeatability between all 10 cycles for all pressure levels. However, it was less accurate at the applied pressure of 72.7 mmHg. The Tactilus exhibited the least repeatable results.

Figure 8b,e,h,k,n shows pressure results obtained from the calibration file. They follow the same change patterns displayed by output voltage readings. Very good agreement between measured pressure readings and applied gold standard pressures of 30.5 and 51.4 mmHg, shown by the dotted lines, was achieved. This could be attributed to matching timings of 30 s for both calibration and measurement. However, for higher pressure of 72.7 mmHg, the least agreement was demonstrated for most of the sensors; the Tekscan Flexiforce and Sensitronics underestimated the pressure and the Interlink and Tactilus overestimated the pressure.

Figure 8c,f,i,l,o shows the accuracy of the sensor pressure readings as a percentage of the gold standard represented by the applied weight. For all sensors, except the Tekscan Flexiforce, pressure reading accuracy was above 85%. The best-performing sensors—Peratech, Sensitronics and Interlink—showed even higher accuracy of 88–97% for all tested pressure values. Again, highest accuracy was achieved at 51.4 mmHg applied pressure level. Measurements at the lowest pressure level of 30.5 mmHg were fairly accurate, with occasional outliers in some cycles.

Drift was not evaluated for dynamic tests because of the short duration of the cycle and inconsistent drift parameter trends within the cycle for most sensors. Instead, average output accuracies with corresponding average of absolute deviations were estimated for three repeats of 10 cycles at 26 s from the start of each cycle (Table 4). Best repeatability was found for the Interlink sensor, ranging from 1.1–1.9%, followed by Peratech 2.1–2.9%, Tactilus 2.1–3.8%, Sensitronics 2.6–6.1% and the least repeatable was the Tekscan Flexiforce at 4.1–7.4%. However, instantaneous variations of 0.85–11% were possible within a single set of 10 dynamic cycles.

## 4. Discussion

Generally an accuracy of 90% is acceptable in compression therapy. For dynamic measurements, only the Peratech, Interlink and Sensitronics sensors satisfy this condition. For static measurements this accuracy was only partially achieved by all sensors, predominantly at medium and higher pressure levels, with some sensors not achieving the required accuracy at all. However, as mentioned earlier, adjusting calibration for longer operating times would significantly improve the accuracy of static measurements and could bring most of the sensors within the required accuracy level. Different calibrations might be needed for short and long-term measurements. Implementing drift correction via post-processing software could further increase the accuracy of the measurements. It is easier to apply calibration corrections to sensors with smaller drift over time. Sensors with larger drifts are slower to respond and would require reconsidering the initial parameter value timing for improved calibration.

The sensors did not consistently display trends of improved accuracy with increased applied pressure. Most of the sensors achieved greatest accuracy at intermediate pressure. However, more testing points are required to establish the pressure at which each sensor is most accurate. Dynamic measurements showed high levels of accuracy, often exceeding 95%, which are explained by matching calibration and measurement timings. Some variations in repeatability and cycle value came to light during dynamic testing and can be dealt with using averages of multiple values for calibration and measurements.

## 5. Conclusions

We studied five commercial flexible resistive pressure sensors and evaluated their suitability for monitoring of compression therapy applications. Sensors were tested at 30.5, 51.4 and 72.2 mmHg of pressure applied via standard weights. For static testing, sensors were subjected to pressure for 8 h. For dynamic testing, 10 cycles of 30-s pressure applications were performed with three repeats for each cycle. In a comprehensive analysis based on the requirements of compression therapy, the Peratech and Sensitronics sensors were found to be suitable for dynamic compression therapy applications, assuming that accuracy of 90% is acceptable. A medium pressure level of 51.4 mmHg resulted in the most accurate readings from most sensors in both static and dynamic tests, with the Peratech being the most accurate sensor under static conditions and Interlink under dynamic conditions, both over 95% accurate. Measurements at 30.5 mmHg were the least accurate, often below the acceptable level and as low as 55%. Accuracy did not increase in line with applied pressure. For sensors exhibiting the lowest drift in output parameters over time, such as the Peratech and Sensitronics, improved accuracy is possible via adjustment of calibration timings. Drift in the output values after 8 h was 0.7–18% for fast responding sensors. Slower-responding sensors, like the Interlink and Tactilus, exhibited drift up to 50% and require longer calibration timings. Repeatability of dynamic measurements was deemed sufficient for compression therapy applications.

## Figures and Tables

**Table 1:** Geometry and sensing properties of evaluated sensors.

Sensor	Manufacturer	Sensing Area	Claimed Operating Range
QTC™ SP 200-10	Peratech Holdco Ltd.	10 mm	0.1–20 N [27]
FSR^®^ 402	Interlink Electronics Inc.	14.7 mm	~0.2–20 N [28]
Half Inch ThruMode FSR	Sensitronics Inc.	12.7 mm	0.3–30 psi [29]
Tactilus^®^	Sensor Products Inc.	12.5 mm	0–400 psi [30]
Flexiforce^®^ A 301	Tekscan Inc.	9.53 mm	0–1 lb [31]

**Table 2:** Applied weights and corresponding pressure.

Disc	Applied Weight (g)	Calculated Pressure (mmHg)
t-pin platform	16.64	17.63
+Weight 1	18.97	20.10
+Weight 2	28.80	30.51
+Weight 3	38.66	40.96
+Weight 4	48.53	51.42
+Weight 5	58.57	62.05
+Weight 6	68.62	72.70
+Weight 7	78.69	83.37
+Weight 8	88.75	94.03

**Table 3:** Average accuracy in static pressure measurements.

Sensor	Accuracy (%)		
	30.5 mmHg	51.4 mmHg	72.7 mmHg
Peratech	83.7	98.1	95.6
Interlink	67.0	74.9	-
Sensitronics	84.3	87.2	84.9
Tactilus	-	92.8	89.5
Tekscan Flexiforce	70.4	96.4	85.8

**Table 4:** Average accuracy in dynamic pressure measurements.

Sensor	Accuracy (%)		
	30.5 mmHg	51.4 mmHg	72.7 mmHg
Peratech	94.8 ± 2.9	96.0 ± 2.1	96.0 ± 2.2
Interlink	97.7 ± 1.1	94.4 ± 1.7	88.3 ± 1.9
Sensitronics	90.8 ± 6.1	94.0 ± 3.1	92.6 ± 2.6
Tactilus	-	87.9 ± 3.8	90.3 ± 2.1
Tekscan Flexiforce	64.1 ± 7.4	89.4 ± 5.0	82.0 ± 4.1
